# Hemostasis and Anti-Inflammatory Abilities of AuNPs-Coated Chitosan Dressing for Burn Wounds

**DOI:** 10.3390/jpm12071089

**Published:** 2022-06-30

**Authors:** Juin-Hong Cherng, Cheng-An J. Lin, Cheng-Che Liu, Jue-Zong Yeh, Gang-Yi Fan, Hsin-Da Tsai, Chun-Fang Chung, Sheng-Der Hsu

**Affiliations:** 1 Graduate Institute of Life Sciences, National Defense Medical Center, Taipei 114, Taiwan; i72bbb@gmail.com; 2 Department and Graduate Institute of Biology and Anatomy, National Defense Medical Center, Taipei 114, Taiwan; u9310318@gmail.com; 3 Department of Biomedical Engineering and Center for Biomedical Engineering in Cancer, Chung Yuan Christian University, Taoyuan 320, Taiwan; chengan_lin@cycu.edu.tw; 4 Department of Physiology and Biophysics, Graduate Institute of Physiology, National Defense Medical Center, Taipei 114, Taiwan; chencheliu2002@gmail.com; 5 Department of Pharmacy, Tri-Service General Hospital, National Defense Medical Center, Taipei 114, Taiwan; yehjuezong@gmail.com; 6 Laboratory of Adult Stem Cell and Tissue Regeneration, National Defense Medical Center, Taipei 114, Taiwan; treker0315@gmail.com (H.-D.T.); lovespring212@gmail.com (C.-F.C.); 7 Division of Traumatology, Department of Surgery, Tri-Service General Hospital, National Defense Medical Center, Taipei 114, Taiwan

**Keywords:** chitosan, AuNPs, burn, wound dressing, healing, inflammation, hemostasis

## Abstract

Burn injuries are a common hazard in the military, as fire is likely to be weaponized. Thus, it is important to find an effective substance to accelerate burn wound healing. This study used chitosan and gold nanoparticles (AuNPs) as wound dressings and investigated their effectiveness in femoral artery hemorrhage swine and rat burn models. Chitosan dressing has significant hemostatic properties compared with gauze. Histological results showed that burn wounds treated with chitosan or AuNP-coated chitosan dressings exhibited more cells and a continuous structure of the epidermis and dermis than those of the control and untreated lesion groups. Furthermore, both chitosan dressings have been shown to positively regulate the expression of genes- and cytokines/chemokines-related to the wound healing process; AuNP-coated chitosan significantly lessened severe sepsis and inflammation, balanced the activities of pro-fibrotic and anti-fibrotic ligands for tissue homeostasis, regulated angiogenesis, and inhibited apoptosis activity, thereby being beneficial for the burn microenvironment. Hence, chitosan alone or in combination with AuNPs represents a prospective therapeutic substance as a burn dressing which might be helpful for burn wound care. This study provides a novel hemostasis dressing for modern warfare that is simple to use by most medical and paramedical personnel handling for burn treatment.

## 1. Introduction

Wars and their hardships have plagued humanity since the dawn of time. Burn in-juries in wartime are ubiquitous threats, and its risks to military personnel during combat are well recognized and have become the most important trauma risk factor during war. Burn injuries are a global public health concern. According to the World Health Organization (WHO), 180,000 people die each year from burns globally, mainly in low- and middle-income countries, and nearly two-thirds of the cases occur in Africa and Southeast Asia [1]. Taiwan’s health welfare department reported the death of 1196 people due to fire accidents between 2007–2017 [2]. Therefore, improving burn treatment technology is important. By controlling the infection, stable repair, reconstruction of wound epithelium, reduction of scar formation, and controlling the pain, the quality of life of burn patients can be improved.

Biomaterials and hydrogel-like materials have been developed to treat burned skin [3]. Chitosan is one of the biomaterials that has attracted significant attention. Chitosan (also known as (1→4)-2-amino-2-deoxy-β-D-glucan) is formed from N-acetyl-D-glucosamine monomers with β-1,4-glycosidic bonds. It is obtained through deacetylation of chitin extracted from crustacean shells [3]. Chitosan is a well-known substance that has antibacterial, anti-inflammatory, hemostatic, and skin regenerative properties, as well as excellent biocompatibility and biodegradability [4,5,6]. Chitosan acts as the first step in the wound-healing process by aiding the cessation of bleeding via promotion of platelet and erythrocyte aggregation and obstruction of fibrin dissolution during the hemostasis phase [3,7]. Wang et al. used hydrogel sheets based on chitosan as a cover for burn wounds to inhibit the growth of bacteria in the inflammation phase, followed by expediting skin proliferation by driving granulation tissue growth in the proliferation stage of wound healing [8]. At this stage, chitosan also accelerates burn wound recovery by attracting inflammatory cells, macrophages, and fibroblasts [9]. Thus, chitosan is a potential substance that can be used to accelerate healing [10].

Marked breakthroughs in nanotechnology for therapeutic applications (nanotherapeutics) efficiently prevent infection and facilitate burn wound healing [11]. Gold nanoparticles (AuNPs) are generally considered potential particles for use in biological application due to their high biocompatibility and physicochemical properties [12]. AuNPs exhibit antibacterial and antioxidant activities. Consequently, AuNPs are important agents in the wound healing process [13,14]. A recent study reported that AuNPs formed nano-bio interactions with skin lipids and were able to open the stratum corneum and penetrate the skin barrier [15]; thus, directly contacting the damaged cells in the wound area. The idea of using AuNP in wound healing therapy is supported by previous findings that some materials, such as cellulose and polysaccharides encapsulated into AuNPs, have been used as dressings to enhance cell proliferation in burn wounds [16,17,18]. Moreover, the addition of metallic nanoparticles, such as AuNPs, to chitosan encourages the improvement of cell adhesion as well as shows more pronounced angiogenesis, fewer inflammatory cells, and well-organized collagen fibers in vivo, which drives better wound repair processes [19,20].

In this study, we aimed to investigate the hemostatic effect of chitosan dressing in a femoral artery hemorrhage swine model and evaluate the underlying mechanisms of burn wound healing and the effects of chitosan or AuNP-coated chitosan dressing treatment in a rat burn model.

## 2. Materials and Methods

### 2.1. Experimental Design

Surgical cotton gauze (sterile, 10 × 10 cm^2^) was purchased from China Surgical Dressings Co. (Taipei, Taiwan) and applied over the hemostatic dressing to fill the wound cavity. Two novel chitosan-based dressings were used in the experimental groups: pure and AuNP-coated chitosan dressings. We used a femoral artery hemorrhage swine model to confirm the hemostatic effect of the chitosan dressing, and a rat burn model was developed to evaluate the underlying mechanisms of burn wound healing following chitosan or AuNP-coated chitosan dressing treatment. All animal experiments were approved by the Institutional Animal Care and Use Committee (swine model = IACUC-15-337; rat model = IACUC-19-147; IACUC-20-210) at the National Defense Medical Center (Taipei, Taiwan).

### 2.2. Gold Nanoparticles (AuNPs)

#### 2.2.1. AuNPs Preparation

The AuNPs were provided by Dr. Cheng-An J. Lin, who provided technical guidance for the use of AuNPs in this study. AuNPs were synthesized using the methods described by Lin et al. [21]. Briefly, using the ligand exchange method, artificial/natural lipoic acid (DL-α-Lipoic Acid, TCI Chemicals, Japan) was oxidized to a covalent bond (thiol, -SH) of a dithiol group, which was coated on the surface of the AuNPs relying on the strong covalent bond force between gold and sulfur. Referring to the one-pot synthesis method proposed by Aldeek et al. [22], gold tetrachloride (Chlo-roauric acid, 2.5 mM, 10 mL) was slowly added to natural lipoic acid (R-α-Lipoic Acid, 1.45 mM, 1 L) at 25 °C and stirred with a homogenizer at 300 rpm for 5 min, and sodium borohydride (50 mM, 20 mL) was slowly added using a titration pump. After the titration was completed, the homogenizer continued to stir at 300 rpm for 15 h. A 1-L solution was concentrated to 20 mL under reduced pressure at 40 °C and was then filtrated using a 10 kDa ultrafiltration centrifuge tube (Amicon^®^ Pro Purification System with 10 kDa Amicon^®^ Ultra-0.5 Device, Millipore, Burlington, MA, USA). Purification was performed by centrifugation at 3500 rpm for 13 min. Finally, fluorescent gold nanoclusters (AuNCs-DHLA) at a concentration of 80 μM surface coated with native lipoic acid were stored at room temperature.

#### 2.2.2. AuNPs Characterization

The optical properties of the AuNPs were measured using a JASCO V 550 UV-Vis spectrophotometer, and the fluorescence spectra of the AuNPs were recorded using a JASCO FP-750 spectrofluorometer. Transmission electron microscopy (TEM) was performed using a Hitachi H-7650 microscope (Hitachi Ltd., Brisbane, CA, USA) at an accelerating voltage of 120 kV to observe the AuNPs’ morphology. Finally, 2% agarose gel electrophoresis was used to examine the chemical properties of the AuNPs using the Bio-Rad horizontal gel electrophoresis system at 75 V.

### 2.3. Chitosan Dressing Preparation

Chitosan dressings were produced using a wet-spinning method. Raw chitosan material (MW = 100 kDa, degree of deacetylation ≥85%) was purchased from Une Shin Trading Co., Ltd. (CAS No. 9012-76-4; New Taipei City, Taiwan). The material was dissolved in 3% (*v*/*v*) and 5% (*w*/*v*) concentration of acetic acid by stirring overnight at 25 °C. The solution was subsequently diluted with methanol to obtain a final concentration of 3% (*w*/*v*), followed by solution filtering through a cloth filter in an ultrasonic bath to remove air bubbles. Further, the solution was injected into a coagulation bath maintained at 40 °C containing a 10% solution of 1 M NaOH in distilled water. The fibers were allowed to form in this medium for overnight, followed by washing several times with distilled water. The fibers were suspended in aq. 2% TWEEN20 for 5 min, followed by soaking in 50, 60, and 70% methanol for 5 min each. The obtained chitosan filaments were thus compressed to drain the absorbed liquid on the mangled machine and were oven-dried at 60 °C in a mold. Finally, the chitosan dressing was sterilized using 25 kGy gamma radiation before use. For chitosan-coated AuNP dressing preparation, a 1 cm × 1 cm chitosan dressing was soaked in AuNPs 30 nm in 100 μL of PBS solution for 10 s and then subjected to air drying.

### 2.4. Femoral Artery Hemorrhage Swine Model

A total of eight crossbred yorkshire pigs (castrated males) weighing 34–37 kg were used in this study. Animals had unlimited access to water and were fasted for 12 h prior to surgery. Before surgery, pigs were intramuscularly injected with buprenorphine (0.025 mg/kg) for analgesia and glycopyrrolate (0.01 mg/kg, i.m.) to reduce saliva secretion and block bradycardia. Pigs were further administered tiletaminezolazepam (Telazol, 4–6 mg/kg, i.m.) and anesthetized with 5% isoflurane in oxygen via a facemask. The tidal volume and ventilation rate were adjusted to maintain the end-tidal carbon dioxide in the range of 38–42 mm Hg. Anesthesia was maintained with 1–2% isoflurane in oxygen via a ventilator. An intravenous catheter was inserted into the ear vein for venous access, and lactated Ringer’s solution was maintained at a rate of 5 mL/kg^−1^/h^−1^.

Furthermore, the right carotid artery was cannulated for blood collection as well as for monitoring blood pressure (systolic, diastolic, and mean) and heart rate throughout the experiment. The femoral artery was identified and partially exposed at a depth of ~5 cm. The overlying abductor muscle was carefully removed to avoid injuries to the adjacent femoral vein and nerve. A total of 2 mL of 2% lidocaine was applied to the artery for 5 min to reduce arterial vasospasm and dilate the artery to its normal size. For wound-site temperature monitoring, a microelectrode was sutured to the muscle adjacent to the vessel, but at least 2.54 cm away from the arteriotomy site, to minimize the interference of the hemostatic treatment. The artery was occluded proximally and distally with vascular clamps and a 6-mm diameter arteriotomy was made on the anterior surface of the artery using a vascular punch (IBC Aortic Punch, Medium, 6.0 mm; International Biophysics Corp., Austin, TX, USA). The vascular clamps were then removed to allow for 45 s free bleed, chitosan or gauze dressing was applied, and the time of hemostasis was recorded manually.

### 2.5. Rat Burn Model

**Figure 1:**
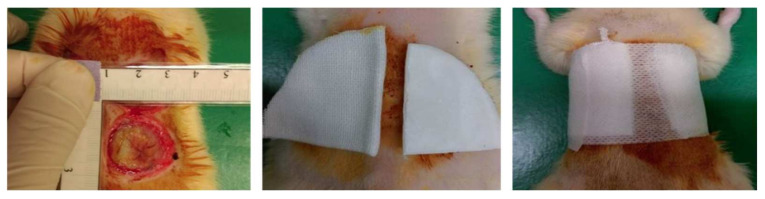
The process of burn injury creation on the rat’s skin.

Thirty-six male Sprague-Dawley rats (250–300 g) were purchased from Bio-LASCO Co. Ltd. (Taipei, Taiwan) for use in this study. Rats were randomly divided into four groups (n = 9): control group, lesion control group, chitosan dressing group, and chitosan-AuNPs dressing group. Rats were given a standard diet ad libitum several days prior to the investigation, and were fasted overnight before any procedures. They were placed in individual cages upon arrival and allowed to acclimatize for a minimum of seven days. Animals were pretreated with ketoprofen (4 mg/g) for pain management. On the day of burn injury induction, rats were sedated by intramuscular injection of Zoletil 50 (20 mg/kg; Verbac, France). The rat’s shaved back and skin were scrubbed with povidone-iodine solution. A full-thickness scald burn was induced in rats by means of a brass block with 4 cm diameter [23] after heating it in a boiling solution until the temperature reached 190 °C, as measured by a thermometer. The brass block was then applied onto the rat’s back parallel to the midline for 20 s to produce third-degree burns. Sixty minutes after burning, the wound was either treated with chitosan dressing or chitosan-AuNPs dressing, or left untreated for the lesion control group (Figure 1). The treated wounds were protected with non-stick cotton and fixed with adhesive dressing to prevent dressing tearing. Iodine solution was used daily to disinfect the wounds. After 7 days, the blood from each group was collected, and the rats were euthanized using lethal doses of CO_2_, followed by burned-skin tissue collection. Skin tissues were fixed for further analysis using 4% paraformaldehyde in PBS.

### 2.6. Histological and Immunohistochemistry Analysis

The fixed rat skin tissues were embedded in paraffin and sectioned at an 8 μm thickness. Samples were stained with hematoxylin and eosin (H&E; Merck Millipore, Darmstadt, Germany) following the manufacturer’s instructions. Stained sections were observed and photographed using an inverted microscope (BX53; Olympus, Tokyo, Japan). Immunohistochemical staining for thrombomodulin (TM), tumor necrosis factor α (TNF-α), inducible nitric oxide synthase (iNOS), arginase-1 (Arg-1), and interleukin-10 (IL-10) (Bioss Antibodies, Woburn, MA, USA) was performed using the ABC method with a Vectastain ABC HRP kit (Vector Laboratories, Burlingame, CA, USA) according to the manufacturer’s instructions. A citrate-based antigen unmasking solution (Vector Laboratories) was used for antigen retrieval, and the primary antibody was used to target macrophages and endothelial cells. The sections were counterstained with Mayer’s hemalum solution (Merck Millipore). Finally, samples were observed and photographed using an Axio Scan Z1 slide scanner (ZEISS). The positively stained area was also semi-quantitatively examined using ImageJ software (NIH, Bethesda, MD, USA).

### 2.7. Prothrombin Time (PT) and Activated Partial Thromboplastin Time (aPTT) Analysis

Blood collected from each group was transferred to vials containing 3.2% (*w*/*v*) sodium citrate. Blood from healthy rats was used as the control. For PT and aPTT analyses, the sera were fed into an automated blood hemostasis analyzer (CS-2100i; Sysmex Corp., Kobe, Japan). Each experiment was performed in triplicate.

### 2.8. RNA Purification and Quantitative Real Time Polymerase Chain Reaction (qRT-PCR)

Total RNA from harvested skin tissues was isolated using the RNeasy Mini Kit (Qiagen, Valencia, CA, USA) under RNase-free DNase digestion. RNA integrity was confirmed using an Agilent 2100 Bioanalyzer. High-capacity cDNA reverse transcription (ABI 4368814, Applied Biosystems, Foster City, CA, USA) was performed. The converted DNA was subjected to qRT-PCR with specific primers for the suppressor of mothers against decapentaplegic (SMAD) 1, SMAD2, iNOS1, Caspase-8, Caspase-3, bcl-2-associated X (BAX), B-cell lymphoma 2 (BCL2), nuclear factor-erythroid 2-related factor 2 (NRF2), Arg1, transforming growth factor beta-1 (TGF-β1), and nuclear factor kappa enhancer binding protein (NF-κB) (1:1000 dilution; all from Santa Cruz Biotechnology, Santa Cruz, CA, USA), using an Applied Biosystems^®^ 7500 Real-Time PCR System (Applied Biosystems) in a 96-well optical plate format. Amplification was performed in 25 μL volume reactions containing Power SYBR Green PCR Master Mix (ABI 4367659, Applied Biosystems), according to the manufacturer’s recommendations. The cycling conditions consisted of a 30 min reverse transcription step at 50 °C, a 2 min denaturation step at 95 °C, and 40 amplification cycles of 15 s at 95 °C and 1 min at 60 °C. PCR-grade water was used as a negative control. The 2^−ΔΔCt^ method was used to calculate relative gene expression, and GAPDH was used as the reference gene for normalization. Each sample was assayed (measured) in triplicates.

### 2.9. Multiplex Growth Factors in Burn Wound

For endothelial growth factor quantification, the MILLIPLEX^®^ Mouse Angiogenesis/Growth Factor Magnetic Bead Panel (Merck Millipore) was used according to the manufacturer’s protocol. Briefly, the burn wound skin of rats was collected, homogenized to extract protein, followed by incubation with antibody-conjugated magnetic beads, including placenta growth factor (PLGF), fibroblast growth factor 1 (FGF-1), vascular endothelial growth factors (VEGF)-C, and VEGF-D, overnight at 4 °C. A Magpix Multiplex Platform (Luminex Corporation, Austin, TX, USA) was employed to read the concentration of the recovered bead complexes, and the median fluorescent values were measured and recorded from a minimum of 80 beads that were wielded for data analysis. Each sample was assayed in triplicates and the mean value of each factor was used for statistical analysis. Milliplex Belysa™ Immunoassay Curve Fitting Software was used to analyze the standard curve and data.

### 2.10. Data Analysis

Data were analyzed using SPSS (version 18.0, SPSS Inc., Chicago, IL, USA) and expressed as the mean ± standard error of the mean. Data were analyzed using one-way analysis of variance (ANOVA) or a two-tailed paired *t*-test. Significant differences between the groups were indicated by * *p* < 0.05, ** *p* < 0.01, and *** *p* < 0.001.

## 3. Results

### 3.1. Hemostasis Analysis

**Figure 2:**
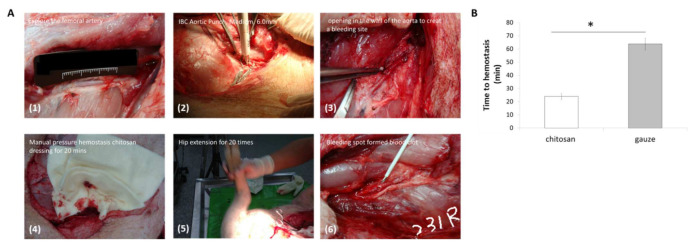
The femoral artery hemorrhage swine model. (**A**) The process of femoral artery hemorrhage creation (the number on the figure shows the order of surgery process); (**B**) Time to hemostasis comparison between chitosan dressing and gauze (* *p* < 0.05).

To investigate the hemostasis capabilities of chitosan dressing, we compared its performance with that of gauze dressing in free-bleed femoral artery hemorrhage in swine; the process of femoral artery hemorrhage creation was shown in Figure 2A. The results showed that chitosan dressing achieved hemostasis significantly faster than gauze dressing, implying the excellent hemostatic characteristics of chitosan (Figure 2B).

### 3.2. The Characterization of AuNPs

**Figure 3:**
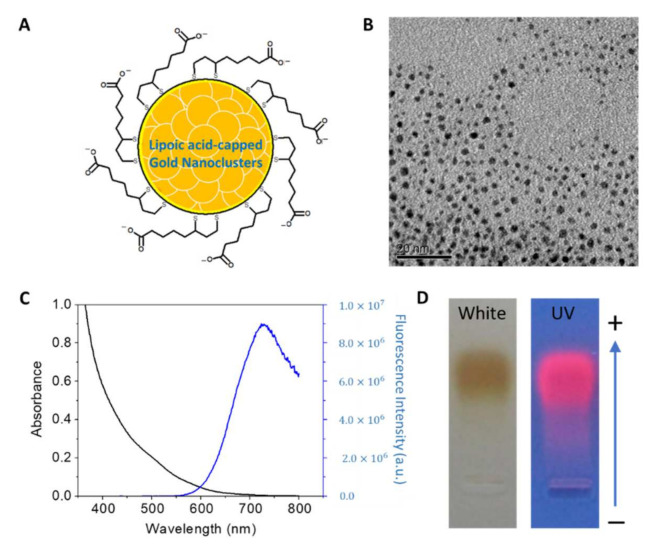
Characterization of gold nanoparticles (AuNPs). (**A**) Illustration of lipoic acid-capped AuNPs containing tens of Au atoms per nanoclusters; (**B**) TEM image of AuNP-DHLA (scale = 20 nm); (**C**) UV-Vis absorption spectra (black line) and emission spectra (blue line, excitation at 490 nm) of AuNP-DHLA; (**D**) Gel electrophoresis photograph of AuNPs in 2% agarose gels under white light and UV light exposure (λex = 365 nm).

In this study, AuNPs were synthesized using the ligand-exchange method with lipoic acid (Figure 3A). Lipoic acids are bidentate capping ligands that form covalent bonds with the thiol group of AuNPs. The synthesized AuNPs (AuNPs-DHLA) were further characterized using different techniques to confirm purity and successfull synthesis. TEM results demonstrated that the AuNPs were monodispersed and not aggregated, with a uniform size ranging between ~1–2 nm (Figure 3B). The UV-vis absorption spectra demonstrated a subtle absorption band around 500 nm (black line), implying no surface plasmon resonance. However, the luminescent spectra displayed a broad band with a peak value of ~720 nm (blue line) (Figure 3C), indicating the successful assembly of gold nanoparticles. Moreover, gel electrophoresis demonstrated that AuNPs exhibited homogeneous negative charges and strong red luminescence (Figure 3D).

### 3.3. Histology and Immunohistochemistry (IHC) Analysis

**Figure 4:**
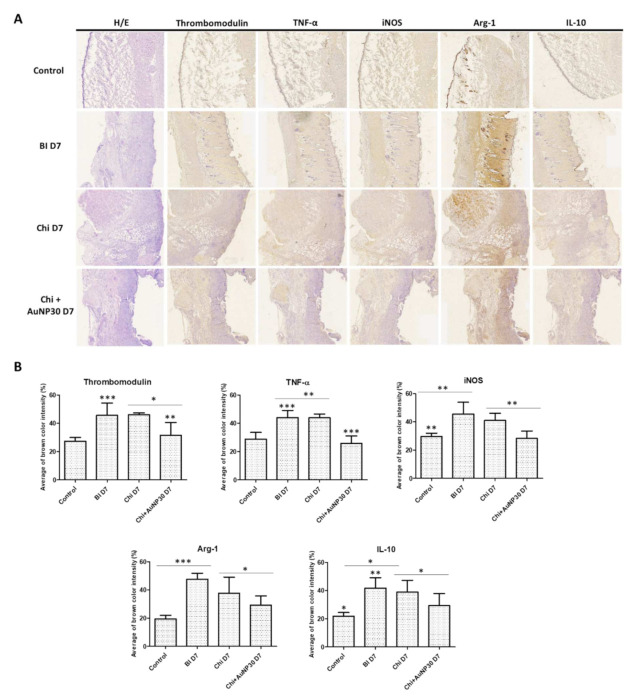
Histological and immunohistochemistry analysis of rat burn wound tissues in the control, lesion, chitosan dressing, and AuNP-coated chitosan dressing groups. (**A**) Skin tissues stained for H&E, TM, TNF-α, iNOS, Arg-1, and IL-10 markers, respectively; (**B**) Semi-quantitative analysis of the relative amounts of brown stained-positive cells with all markers amongst all groups. Control = normal control group; BI D7 = lesion control group day 7 post-wounding; Chi D7 = chitosan dressing treatment day 7 post-wounding; Chi + AuNP30 D7 = AuNP-coated chitosan dressing treatment day 7 post-wounding; * *p* < 0.05, ** *p* < 0.01, *** *p* < 0.001.

Using the swine femoral artery hemorrhage model, we revealed that chitosan dressing exhibited outstanding hemostatic properties compared to gauze. Further, we investigated the chitosan–AuNPs dressing and compared their effectiveness to that observed in the chitosan dressing alone, control, and lesion control groups of the rat burn model to explore the capabilities of chitosan dressing in treating wounds using this beneficial combination. Histological analysis of H&E staining showed that burn wounds treated with chitosan dressing or AuNP-coated chitosan dressing revealed more cells, granulation tissue formation in the underlying dermis, and intact normal histopathological structure of the epidermis and dermis compared to those observed in the control and untreated lesion groups (Figure 4A). Moreover, the IHC results demonstrated that the expression of brown stained-positive cells in burn wounds for all markers in the chitosan dressing and AuNP-coated chitosan dressing groups was less than that observed in the control and untreated lesion groups, which was further confirmed by semi-quantitative analysis using ImageJ software (Figure 4B). These results indicate that both chitosan dressing treatments significantly reduce the expression of TM, TNF-α, iNOS, Arg-1, and IL-10 when compared with the untreated lesion. In addition, the untreated lesion group showed significantly enhanced TM, TNF-α, iNOS, Arg-1, and IL-10 expression compared with that observed in the control group.

### 3.4. Blood Coagulation Analysis in Burn Wound

**Figure 5:**
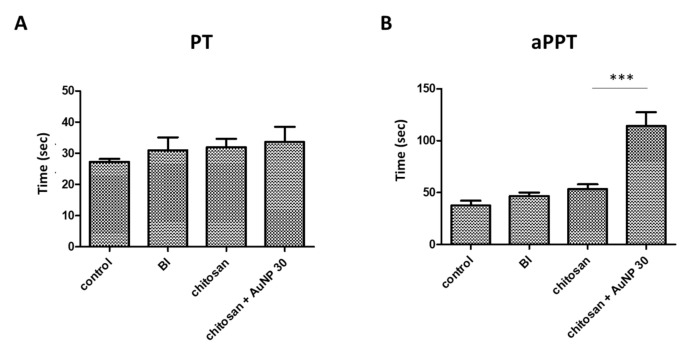
The effects of treatments on (**A**) prothrombin time (PT) and (**B**) activated partial thromboplastin time (aPTT). BI = lesion control group; *** *p* < 0.001.

PT and aPTT tests were performed to evaluate the ability of the blood to form clots over time as a function of treatment. We measured the amount and function coagulation factors, which are important for proper blood clot formation. As shown in Figure 5A, there was no significant difference in the PT times among all groups; thus, indicating that both treatments had no significant effect on the extrinsic coagulation pathway in the burn wound. However, the aPTT time results demonstrated that the AuNP-coated chitosan dressing treatment showed the longest time among all groups (Figure 5B), implying its significant influence on the internal coagulation mechanism, which might stimulate the intrinsic coagulation pathway.

### 3.5. Fibrotic Formation Analysis in Burn Wound

**Figure 6:**
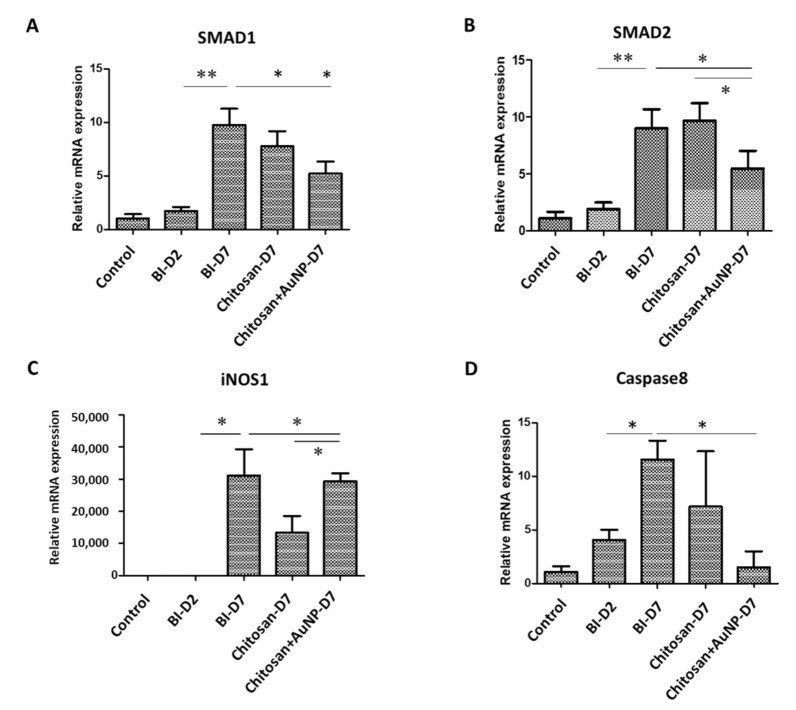
The relative mRNA expression level of several fibrotic biomarkers in rat burn wound tissues from the control, lesion, chitosan dressing, and AuNP-coated chitosan dressing groups. (**A**) SMAD1; (**B**) SMAD2; (**C**) iNOS1; (**D**) Caspase-8. Control = normal control group; BI D2 = lesion control group day 2 post-wounding; BI D7 = lesion control group day 7 post-wounding; Chi D7 = chitosan dressing treatment day 7 post-wounding; Chi + AuNP30 D7 = AuNP-coated chitosan dressing treatment day 7 post-wounding; * *p* < 0.05, ** *p* < 0.01.

We investigated the levels of gene-related fibrotic formation, including SMAD1, SMAD2, iNOS1, and Caspase-8 in rat burn injury (Figure 6). Overall, we observed that the levels of SMAD1, SMAD2, and Caspase-8 significantly decreased in both chitosan dressings treatments compared to those in the untreated lesion group (Figure 6A,B,D), with the lowest levels observed in the group with AuNP-coated chitosan dressing. Both chitosan dressing treatments also significantly suppressed the level of iNOS1 compared to that associated with the untreated lesion group (Figure 6C); however, the iNOS1 level in the AuNP-coated chitosan dressing treatment was significantly higher than that in the chitosan dressing treatment alone.

### 3.6. Angiogenesis Analysis in Burn Wound

**Figure 7:**
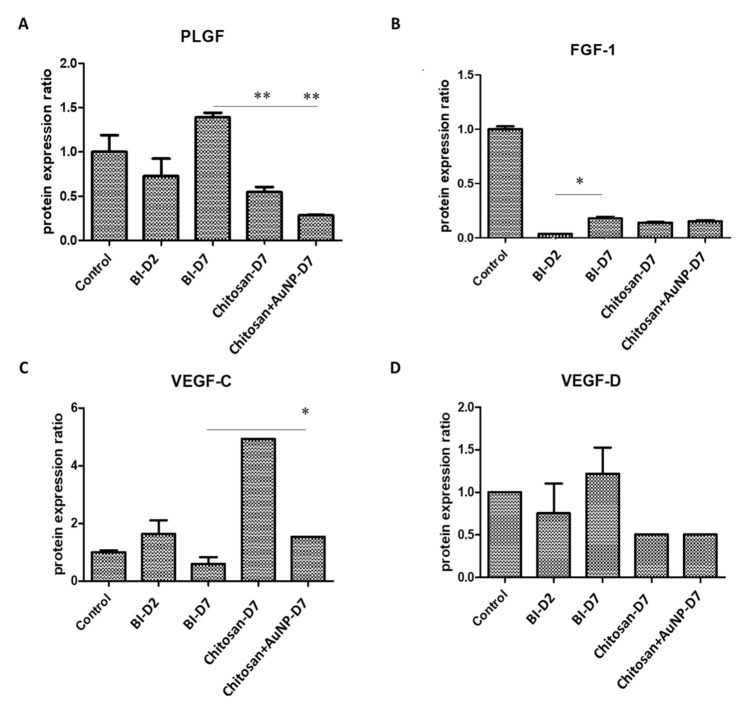
The protein expression of several angiogenesis biomarkers in rat burn wound tissues in the control, lesion, chitosan dressing, and AuNP-coated chitosan dressing groups. (**A**) FLGF; (**B**) FGF-1; (**C**) VEGF-C; (**D**) VEGF-D. Control = normal control group; BI D2 = lesion control group day 2 post-wounding; BI D7 = lesion control group day 7 post-wounding; Chi D7 = chitosan dressing treatment day 7 post-wounding; Chi + AuNP30 D7 = AuNP-coated chitosan dressing treatment day 7 post-wounding; * *p* < 0.05, ** *p* < 0.01.

We investigated the levels of gene-related angiogenesis factors, including PLGF, FGF-1, VEGF-C, and VEGF-D in rat burn injury (Figure 7). Overall, we discovered that the levels of all markers were suppressed in both chitosan dressings treatments compared to those in the untreated lesion and control groups (Figure 7A–D). In particular, the AuNPs-coated chitosan dressing treatment significantly decreased the level of PLGF (Figure 7A) and VEGF-C (Figure 7C).

### 3.7. Inflammation Analysis in Burn Wound

**Figure 8:**
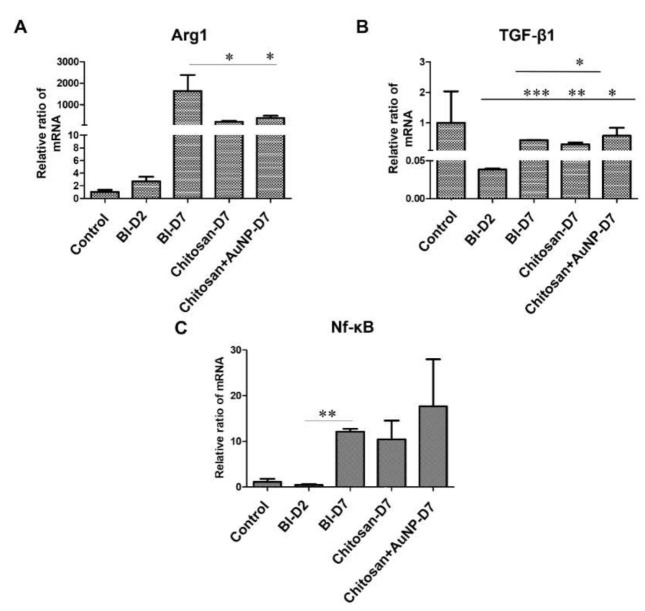
The relative mRNA expression level of several inflammation biomarkers in rat burn wound tissues from the control, lesion, chitosan dressing, and AuNP-coated chitosan dressing groups. (**A**) Arg1; (**B**) TGF-β1; (**C**) Nf-κB. Control = normal control group; BI D2 = lesion control group day 2 post-wounding; BI D7 = lesion control group day 7 post-wounding; Chi D7 = chitosan dressing treatment day 7 post-wounding; Chi + AuNP30 D7 = AuNP-coated chitosan dressing treatment day 7 post-wounding; * *p* < 0.05, ** *p* < 0.01, *** *p* < 0.001.

To observe inflammation in the burn wound area, the levels of gene-related inflammatory responses, including Arg1, TGF-β1, and NF-κB, were measured (Figure 8). Our results showed that both chitosan dressing treatments significantly suppressed the level of Arg1 when compared to that observed in the untreated lesion group (Figure 8A), whereas AuNP-coated chitosan dressing treatment induced a significant decrease in the TGF-β1 level (Figure 8B) when compared to the effects of chitosan dressing treatment and the levels observed in the untreated lesion group (Figure 8B). Moreover, the level of NF-κB was higher in both chitosan dressing treatments; however, no significant difference was found between the groups (Figure 8C).

### 3.8. Apoptosis Analysis in Burn Wound

**Figure 9:**
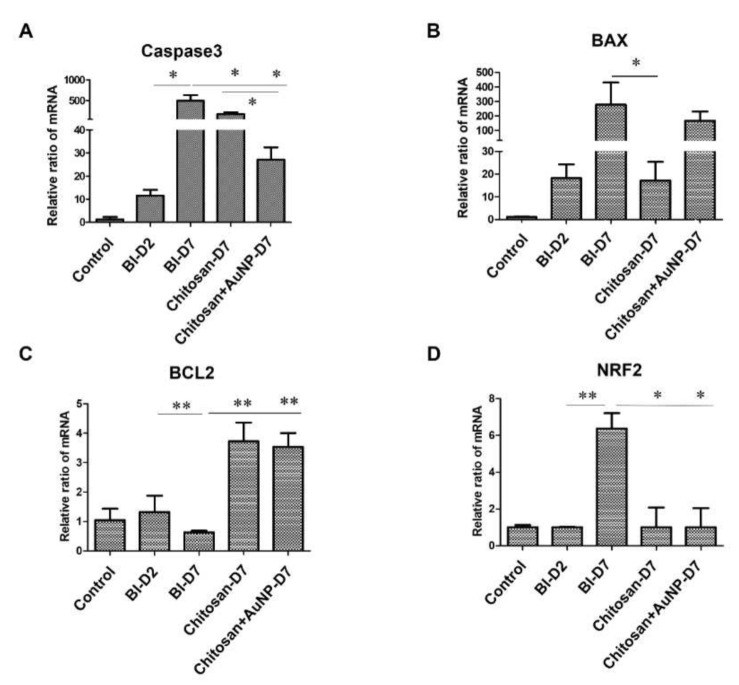
The relative mRNA expression level of several apoptosis biomarkers in rat burn wound tissues from the control, lesion, chitosan dressing, and AuNP-coated chitosan dressing groups. (**A**) Caspase-3; (**B**) BAX; (**C**) BCL2; (**D**) NRF2. Control = normal control group; BI D1 = lesion control group day 1 post-wounding; BI D7 = lesion control group day 7 post-wounding; Chi D7 = chitosan dressing treatment day 7 post-wounding; Chi + AuNP30 D7 = AuNP-coated chitosan dressing treatment day 7 post-wounding; * *p* < 0.05, ** *p* < 0.01.

We investigated the expression levels of apoptosis-related genes Caspase-3, BAX, BCL2, and NRF2 in the rat burn injury model (Figure 9). Our data revealed that chitosan and AuNP-coated chitosan dressing treatment significantly suppressed the levels of Caspase-3 and NRF2 compared with those in the untreated lesion group (Figure 9A,D). Furthermore, the chitosan dressing treatment significantly decreased the level of BAX compared with that observed in the untreated lesion group (Figure 9B); however, AuNP-coated chitosan dressing treatment demonstrated a contrasting tendency, although there was no significant difference. Nevertheless, both chitosan dressing treatments significantly increased the level of BCL2 compared to the those observed in the untreated lesion and control groups (Figure 9C).

## 4. Discussion

Burns are the most common and traumatic injuries in war. Hence, effective wound management is required to significantly improve the survival rate and prognosis of burn patients. In this study, we investigated the effectiveness of chitosan-based dressings in a swine femoral artery hemorrhage model to confirm the hemostatic effect of chitosan dressing, as well as in a rat burn model to evaluate the mechanisms underlying chitosan dressing effects on burn wound healing. Our results demonstrated the potential of chitosan-based dressing as a burn therapeutic. Based on our previous study [24], a porcine femoral artery hemorrhage model was used to examine the hemostatic ability (Figure 2B) of the developed chitosan dressing, with a superior time to cessation of bleeding. Chitosan has positively charged amino groups along its molecular chains, along with cationic moieties such as NH^3+^ that form metal cation–chitosan complexes, promoting hemostasis [25,26]. In this study, we used a AuNP coat (synthesized using the ligand exchange method with lipoic acid) on a chitosan dressing in a rat burn model. Characterization of the synthesized AuNPs revealed the successful assembly of AuNPs with uniform sizes ranging between ~1–2 nm with homogeneous negative charges as well as strong red luminescence, which is in agreement with the quality metrics used in previous reports [27,28]. AuNPs have high reducing ability due to their small size, which may enhance NH^+^ activation. We further performed PT and aPTT time analyses, and the results revealed that AuNP-coated chitosan dressing treatment had the highest aPTT time among all groups, while there was no significant difference in the PT time (Figure 5). This indicates that AuNPs were likely to bind coagulation factors and prolong the aPTT. Nevertheless, aPTT accounts for only a small amount of thrombin formed during acute coagulation.

The immune response to the presence of AuNPs during wound repair has rarely been discussed. Histological results revealed that both chitosan and AuNP-coated chitosan dressing treatment resulted in more keratinocytes, granulation tissue formation in the underlying dermis, and intact histopathological structure of epidermis and dermis in burn wounds, highlighting their benefits for the burn wound microenvironment. Furthermore, both chitosan dressing treatments, especially that supplemented with AuNPs, significantly reduced the expression of TM, TNF-α, iNOS, Arg-1, and IL-10 (Figure 4). TM plays a key role in maintaining homeostasis of the intravascular mechanism due to its anticoagulant and anti-inflammatory properties [29]. TM also serves as a prospective biomarker of endothelial injury, as it can be produced through proteolytic cleavage by leukocyte-derived proteases and metalloproteases (MMPS) that are circulated in sepsis and inflammatory states; thus, allowing the TM level increase several-fold under trauma conditions [30,31,32]. The decreased TM levels associated with the chitosan and AuNP-coated chitosan dressing treatments indicate that the treatments might have lessened severe sepsis and inflammation in burn wounds compared with that observed in the untreated lesion group. In addition, inflammatory mediators, such as TNF-α and IL-10, were shown to be elevated post-burn and involved in the inflammation process [33,34]. Inappropriate production or persistent activation of TNF-α leads to various diseases and delayed wound recovery [35]. Additionally, the TNF-α/IL-10 ratio has been previously used as a biomarker for burn injury severity and infection [36]. Hence, the decrease in TNF-α and IL-10 expression after dressing treatments suggests that chitosan and AuNPs could balance the inflammatory response, thereby promoting proper proliferation of endothelial cells and burn wound recovery [33]. Macrophage activation was significantly elevated in the post-injury period, and its transmembrane signaling system was in a state of continuous activation [37]. Burn wounds induce excessive production of inflammatory cytokines and immunosuppressive factors, resulting in a hyperinflammatory response and cellular immunosuppression. This is an important initiating factor that leads to immune dysfunction following burns. The M1 macrophage marker is iNOS, and M2 macrophage marker is Arg1. Our data indicated downregulated M1 and M2 markers following chitosan treatment, particularly in the AuNP-coated chitosan group. Hence, both chitosan and AuNPs proved to be important substances for wound dressing by regulating the inflammatory response during burn wound healing.

Fibrosis is a natural process during wound healing and is meant to restore tissue shape and function; however, it can cause scarring and dysfunctional tissue if the healing process is disrupted by pathological conditions [38]. Both SMADs play major roles in fibrosis scarring. TGF-β1 has been shown to influence the SMAD2/3 signaling pathway to increase connective tissue deposition and mediate fibroblast to myofibroblast transformation and promote wound enclosure [38]. SMAD2/3 is also involved in the regulation of profibrotic genes such as collagens, integrins, and matrix MMPS. Thus, SMAD2/3 serves as positive feedback to predispose wound repair together with TGF-β1 [39]. In contrast, SMAD1/5/8 impaired TGF-β1 mediated fibrosis at the wound site and increased BMP/SMAD1/5/8 antifibrotic activity [40]. In our results, both chitosan dressings significantly decreased the SMAD1 and SMAD2 levels in comparison with those in the untreated lesion group, thus balancing the activities of the pro-fibrotic and anti-fibrotic ligands and warranting tissue homeostasis (Figure 6). In addition, NO production is mediated by iNOS, which is independently regulated by intracellular calcium elevation [41]. The significant and slight decrease in iNOS expression following chitosan and AuNP-coated chitosan dressing treatments, indicates that chitosan and AuNPs may have modulatory effects that could be beneficial in reducing inflammatory or pathogenic responses [42]. Additionally, chitosan and AuNPs might affect wound healing by balancing the TNF-α/IL-10 ratio but not NO production [33,43]. Alternatively, Caspase-8 is defined as an initiator caspase for apoptosis activated by TNF-α and the TRAIL superfamily ligands. Our results are in agreement with those presented by Lee et al., who showed that Caspase-8 was downregulated in the restored-thickened epidermis after injury but remained unchanged in normal skin, suggesting that inception of Caspase-8 expression was not implicated in epidermal morphogenesis, but controlled epidermal integrity [41].

During the wound healing process, angiogenesis occurs through the invasion of capillary radiates in the fibrin/fibronectin-rich wound clot, forming a microvascular network in granulation tissue [44]. This process is crucial and involves some important genes such as PLGF, FGF, and VEGF. Although angiogenic factors are crucial in wound healing, the characteristics of burn wounds prone to granulation tissue formation must be controlled in clinical setting. Our results indicate that treatment with both chitosan and chitosan + AuNPs stabilized the expression level of the FGF protein family (Figure 7), thus its expression was not significantly different compared to that on the seventh day post-burn [45]. In addition, VEGFs are considered common significant therapeutic tools for their proangiogenic effects in wound healing [46,47]. Mammalian VEGF consists of VEGF, VEGF-B, VEGF-C, VEGF-D, and PlGF, which act as key regulators of vasculogenesis, angiogenesis, lymphangiogenesis, and vascular permeability [48]. In our study, we discovered that VEGF-C was significantly enhanced by chitosan treatment which induced persistent angiogenesis and recruited inflammatory cells in the wound healing process via VEGFR-3 [47]. However, the significant decrease in VEGF-C levels in the non-treated animals on day 7 compared to day 2 post-injury was possibly due to the short half-life of VEGF. This undesirable effect is typically observed at high systemic levels. Thus, the binding of VEGF with other proteins in the cells or probably with chitosan and AuNPs might prolong its life span and support its molecular function in wound healing [11,49,50]. The stable VEGF-C may assist the acceleration of leukocyte migration into the wounds, thereby enhancing the wound healing process [51]. Similarly, VEGF-D also promotes lymphangiogenesis and angiogenesis via VEGFR-3 [52,53]. Surprisingly, the inhibition of VEGF-D mRNA levels by the application of both chitosan and chitosan + AuNPs was required because VEGF-D deficiency leads to faster wound closure [54].

Similar to the IHC results, in the chitosan and AuNP-coated chitosan groups, the excessive production of inflammatory cytokines such as Arg-1 and TGF-β was downregulated on day 7 post-injury (Figure 8). Arg-1 was dynamically expressed during wound healing and exhibited high expression on day 7 post-injury but decreased significantly following chitosan and AuNP treatments [55]. The upregulation of Arg-1 during seven days post-burn can be considered a transition sign from a pro-inflammatory extracellular environment to a matrix deposition phase [56]. TGF-β1 has been shown to greatly affect cell types that are implicated in all stages of wound healing [57]. For instance, it activates mitogen-activated protein kinases, phosphoinositide 3-kinase, and the Rho family of guanosine triphosphatases to regulate cell growth and apoptosis [58], and is involved in wound healing by regulating SMAD-dependent and independent pathways [59,60]. Our results revealed that TGF-β1 expression was significantly decreased in the chitosan + AuNP group, suggesting that chitosan + AuNP treatment was able to avoid overexpression of TGF-β1. Previous studies have reported that excessive and prolonged TGF-β1 expression at the wound site is associated with delayed wound healing [61], while a minimal amount of TGF-β1 signaling is required for proper wound healing [62]. In contrast, NF-κB levels were significantly increased after chitosan dressing treatment. This is in agreement with previous studies that showed NF-κB to be involved in corneal wound healing [63], scratch injury [64], and cutaneous wound healing [65,66].

In wound healing, apoptosis is key for removing inflammatory cells and promoting scar formation [67]. One important gene involved in this process is Caspase-3. It is a cysteine-aspartic acid protease that is activated by Caspase-8 and is responsible for initiating apoptosis. This process, followed by cleavage of the pro-apoptotic BAX protein, initiates the intrinsic pathway of apoptosis (IPA) [67,68]. Hence, our results are in line with the aforementioned claims that Caspase-3 and BAX mRNA levels were elevated in the burn injury group but decreased after chitosan and chitosan + AuNP treatment. This suggests that chitosan and AuNPs can inhibit apoptosis by impairing Caspase-3 and BAX expression [69,70]. Moreover, the balance between pro-apoptotic proteins (Bax) and anti-apoptotic proteins (Bcl-2) is considered crucial in the apoptosis regulation. Bax is involved in apoptosis by translocating from the cytoplasm to the mitochondria, which promotes cytochrome c release, while Bcl-2 inhibits this mechanism [69]. Therefore, the elevated Bcl-2 expression associated with chitosan and chitosan + AuNPs treatments indicates that these compounds are promising for use in wound healing due to their anti-apoptotic activity (Figure 9). The NRF2 transcription factor plays an important role in mediating the cellular stress response [71], including the regulation of several ROS-detoxifying enzymes, antioxidant proteins, and drug transporters [72,73,74]. As NRF2 is responsible for protecting cells from ROS during inflammation [71], NRF2 expression was indeed increased after tissue damage along with ROS production; however, following chitosan and chitosan + AuNPs treatments, the NRF2 levels decreased as the burn wound healing progressed. The purpose of this study was to investigate the effect of chitosan on burn wounds within a short period of time (seven days). This is also a limitation of the present study. One of the notable findings of our study is that cell apoptosis was inhibited by AuNP-coated chitosan dressing treatment. These results should be considered for long-term wound healing as longer observations in animal models can provide sufficient evidence to assess the long-term potential of this treatment.

## 5. Conclusions

In summary, our results suggest that chitosan and its combination with AuNPs are promising candidates as burn dressing treatment. Due to their ability to positively regulate the expression of several genes and cytokine/chemokine-related wound healing processes, including fibrotic formation, angiogenesis, inflammation, and apoptosis, as well as hastening the time to hemostasis compared with gauze treatment, it is possible that chitosan combined with AuNPs could beneficially accelerate the inflammatory and proliferative phases of burn injury healing process. Based on these findings, war wound dressings can be developed and applied in trauma ICUs, replacing or complementing the already available burn dressings.

## Data Availability

Data were available upon request.
